# *LRRC3B* Polymorphisms Contributed to Breast Cancer Susceptibility in Chinese Han Population

**DOI:** 10.3389/fonc.2021.657168

**Published:** 2021-06-10

**Authors:** Yuxin Wang

**Affiliations:** Queen Mary School, Nanchang University, Nanchang, China

**Keywords:** breast cancer, *LRRC3B*, polymorphisms, susceptibility, case–control

## Abstract

**Purpose:**

*LRRC3B* gene, as a tumor suppressor gene was involved in the development and progress of breast cancer (BC). However, the effect of *LRRC3B* polymorphisms on BC has rarely been reported. In the study, we aimed to evaluate the relation between *LRRC3B* variants and BC risk.

**Methods:**

Among 563 BC patients and 552 healthy controls, ten single-nucleotide polymorphisms (SNPs) in *LRRC3B* were genotyped by Agena MassARRAY. Odds ratios (ORs) and 95% confidence interval (CI) were calculated using logistic regression model.

**Results:**

Our study demonstrated that rs1907168 polymorphism (heterozygous: OR = 0.71, *p* = 0.017) was related to the reduced risk of BC in the overall. In stratified analyses by age, rs1907168 was associated with the decreased (heterozygous: OR = 0.53, *p* = 0.002) while rs78205284 (homozygote: OR = 2.83, *p* = 0.034) increased BC susceptibility among the population at age ≤51 years. Rs6551122 (recessive: OR = 0.51, *p* = 0.028) and rs12635768 (homozygote, OR = 0.36, *p* = 0.023) polymorphisms were related to the smaller BC tumor size (<2 cm). In addition, rs112276562 (heterozygote OR = 0.56, *p* = 0.002), rs6551122 (heterozygote OR = 0.63, *p* = 0.016), and rs73150416 (heterozygote OR = 0.57, *p* = 0.005) variants contributed to the lower incidence of PR-positive BC. Moreover, rs6788033 was associated with a lower expression level of Ki-67 (log-additive: OR = 0.68, *p* = 0.024). Furthermore, we found an association of ‘GATT’ haplotype with an increased risk for BC. In addition, *LRRC3B* gene was down-regulated in BC tumor and had a poor prognosis in BC in *in silico* analysis.

**Conclusion:**

Our study firstly found *LRRC3B* SNPs contributed to the risk of BC, suggesting *LRRC3B* variants might help to predict BC progression.

## Introduction

Breast cancer (BC) is the most commonly diagnosed malignant tumor and the primary cause of cancer death among women globally (1). In China, 268,600 new cases of BC were diagnosed and there were 69,500 deaths, and its morbidity and mortality rates have been increasing annually (2). BC is a multi-factorial, polygenic disease resulting from genetic, endocrine, and environmental factors (3). Age, serum hormone levels, family history, and environmental factors have been identified as potential contributors to BC susceptibility (4). Moreover, genetic factors were the major drivers in the genesis of BC (5–7).

Leucine-rich repeat-containing 3B (LRRC3B), also named LRP15, is one of the transmembrane proteins having an evolutionarily conserved LRR-domain (8). LRR-containing proteins participated in protein interaction, cell adherence, DNA repair, gene recombination, and so on (9). LRRC3B protein has been reported to regulate the DNA damage and repair pathways and cell cycle progression (10). Additionally, *LRRC3B* is down-regulated in non-small-cell lung, head and neck, gastric and breast cancers (11–14), suggesting *LRRC3B* involvement in carcinogenesis. Abnormal expression of *LRRC3B* in BC was associated with the development and prognosis of BC (13). In addition, *LRRC3B* also served an important role in BC recurrence and metastasis (15). Genetic polymorphisms may affect its gene expression, thus is associated with the occurrence of disease. In spite of many researchers’ reports on the role of *LRRC3B* expression in BC pathogenesis, the possible effects of *LRRC3B* polymorphisms on BC have not been studied.

Here, we investigated the frequency distribution of *LRRC3B* polymorphisms and the potential relationship of *LRRC3B* polymorphisms with BC susceptibility in Chinese Han females.

## Materials and Methods

### Study Subjects

A total of 1,115 unrelated Chinese Han female subjects (563 BC patients and 552 healthy controls) were enrolled from the Shaanxi Provincial Cancer Hospital. BC patients were newly diagnosed and were confirmed by histopathology based on the WHO BC classification. The blood samples of patients were collected before chemotherapy, radiotherapy, or endocrine therapy. Patients had no other cancer or other systemic inflammatory disease. In the meantime, age-matched healthy individuals who had a checkup at the hospital were recruited as controls. The controls had no mammary tumors by recent clinical and mammographic examinations, no cancer history, no BC family history, and no mammary diseases. The study was approved by the Nanchang University and in accordance with the 1964 Helsinki Declaration. Written informed consent was obtained.

### *In Silico* Analysis

Prediction of *LRRC3B* SNPs’ possible function was performed with HaploReg v4.1 online tool (https://pubs.broadinstitute.org/mammals/haploreg/haploreg.php). With the purpose to assess *LRRC3B* mRNA expression in BC, GEPIA database was used. Kaplan–Meier plotter was used to evaluate the prognosis of *LRRC3B* expression in BC.

### Data Collection and DNA Extraction

Questionnaires and medical records were used to gather the demographic and clinical features. The following data were recorded: age, estrogen receptor (ER), progesterone receptor (PR), human epidermal growth factor receptor 2 (HER2) and Ki-67 status, clinical stage, tumor size, and tumor lymph node invasion. After interview, peripheral blood (5 ml) was gathered into EDTA-coated tubes from patients and healthy controls. Genomic DNA was isolated using the GoldMag genomic DNA purification kit (GoldMag Co. Ltd., Xi’an, China).

### Genotyping of SNPs

We obtained the physical position of the *LRRC3B* gene on chromosome 3:26664297-26752267 through the e!GRCh37 (http://asia.ensembl.org/Homo_sapiens/Info/Index) database. In the VCF to PED Converter window (http://grch37.ensembl.org/Homo_sapiens/Tools/VcftoPed), we entered the gene location, selected the CHB and CHS population, and downloaded the “ped” file and “info” file for the variations of *LRRC3B*. Using Haploview software, we selected tagSNPs based on HWE >0.01, MAF >0.05, Min Genotype >75%, and Tagger r^2^ >0.8. Combining MassARRAY primer design software, HWE >0.05, MAF >0.05, and the call rate >95% in our study population, ten candidate SNPs (rs112276562, rs6790894, rs6551121, rs6551122, rs1907168, rs73150416, rs12635768, rs6551130, rs78205284 and rs6788033) in *LRRC3B* were randomly selected. *LRRC3B* SNPs were genotyped by MassARRAY platform (Agena Bioscience, San Diego, CA, USA) (16, 17). The primers were shown in **Supplementary Table 1**. About 10% of the samples were randomly selected and genotyped in duplicate for quality control, and the concordance was 100%.

### Data Analysis

Student’s t test was performed to evaluate the age distribution. Hardy–Weinberg equilibrium (HWE) was tested by a goodness-of-fit χ^2^ test in controls. Genotype frequencies of *LRRC3B* SNPs between patients and controls were analyzed by χ^2^ test. Odds ratios (ORs) and 95% confidence interval (CI) adjusted age were calculated, and haplotype frequencies were estimated using PLINK software. False-positive report probability (FPRP) analysis was used to evaluate the noteworthy associations of the significant findings (18, 19). We set 0.2 as the FPRP threshold and assigned a prior probability of 0.1 for an association with genotypes under investigation. Pairwise linkage disequilibrium (LD) was analyzed using Haploview software (version 4.2). Multifactor dimensionality reduction (MDR) (version 3.0.2) was performed to evaluate the SNP–SNP interactions in the risk of BC. *p*-values <0.05 were considered significant, whereas a value of corrected *p <*0.05/10 was considered significant after Bonferroni correction.

## Results

The features of all subjects were summarized in **Table 1**. 563 BC patients (52.05 ± 9.81 years) and 552 healthy controls (51.88 ± 9.85 years) participated in the present study. No significant difference of age distribution between cases and controls (*p* = 0.767) was found. Most patients had tumor size >2 cm (56.0%), stage I/II (64.8%), lymph node invasion (48.8%), positive hormone receptor status such as ER+ (67.1%), PR+ (60.6%), or HER2+ (48.5%) and positive Ki67 (64.8%).

**Table 1:** Characteristics of patients with breast cancer and controls.

Characteristics		Cases (n = 563)	Controls (n = 552)	*p*
**Age**	Mean ± SD (years)	52.05 ± 9.81	51.88 ± 9.85	0.767
	**>51**	297 (52.8%)	295 (53.4%)	
	**≤51**	266 (47.2%)	257 (46.6%)	
**Tumor size (cm)**	≤2 cm	107 (19.0%)		
	>2 cm	315 (56.0%)		
	Missing	141 (25.0%)		
**Stage**	I–II	365 (64.8%)		
	III–IV	162 (28.8%)		
	Missing	36 (6.4%)		
**Lymph node invasion**	No	260 (46.2%)		
	Yes	275 (48.8%)		
	Missing	28 (5.0%)		
**ER status**	Negative	161 (28.6%)		
	Positive	378 (67.1%)		
	Missing	24 (4.3%)		
**PR status**	Negative	212 (37.7%)		
	Positive	341 (60.6%)		
	Missing	10 (1.8%)		
**HER2 status**	Negative	91 (16.2%)		
	Positive	273 (48.5%)		
	Missing	199 (35.3%)		
**Ki67**	Negative	132 (23.4%)		
	Positive	365 (64.8%)		
	Missing	66 (11.7%)		

ER, estrogen receptor; RP, progesterone receptor; HER2, human epidermal growth factor receptor 2.

According to the GEPIA database, *LRRC3B* gene was down-regulated in BC (*p* < 0.01, **Supplementary Figure 1**). Kaplan–Meier plotter showed high expression of *LRRC3B* was related to better BC overall survival (HR = 0.83, 95% CI: 0.71–0.97, *p* = 0.017, **Supplementary Figure 2**). We speculated that *LRRC3B* is a tumor suppressor gene, but more convincing studies are needed to validate the conclusions because these clinical data are from the database.

The MAFs of *LRRC3B* SNPs were more than 0.05 (**Supplementary Table 2**). No deviation from the HWE for the polymorphisms examined was observed in the genotype distribution of the controls (*p* > 0.05), suggesting these subjects could represent the general population. In addition, *LRRC3B* variants were related to enhancer histone, changed motifs, and GRASP QTL hits in *in silico* analysis.

We performed allele and genetic model analyses of ten *LRRC3B* SNPs in the BC cases and control subjects. Genotype frequency distribution of SNPs was shown in **Table 2** and **Supplementary Table 3**. The distribution of A and T alleles of *LRRC3B* rs1907168 polymorphism was 84.6 and 15.4% in controls and 87.7 and 12.3% in patients. An association between T allele and BC risk (OR = 0.77, 95% CI: 0.61–0.99, *p* = 0.037) was found. Similarly, rs1907168 had a decreased risk of BC (heterozygous: OR = 0.71, 95% CI: 0.54–0.94, *p* = 0.017; dominant: OR = 0.72, 95% CI: 0.55–0.95, p = 0.019; and log-additive: OR = 0.76, 95% CI: 0.59–0.97, *p* = 0.030). However, no significant association was found for other SNPs (**Supplementary Table 3**).

**Table 2:** Relationships between *LRRC3B* rs1907168 polymorphism and breast cancer risk.

Model	Genotype	Case	Control	Adjusted by age and gender	
				OR (95%CI)	*p*
Allele	A	987	929	1.00	**0.037**
	T	139	169	**0.77 (0.61–0.99)**	
Genotype	AA	432	388	1.00	
	AT	123	153	**0.71 (0.54–0.94)**	**0.017**
	TT	8	8	0.90 (0.33**–**2.42)	
Dominant	AA	432	388	1.00	**0.019**
	AT-TT	131	161	**0.72 (0.55–0.95)**	
Recessive	AA-AT	555	541	1.00	0.963
	TT	8	8	0.98 (0.36**–**2.62)	
Log-additive	---	---	---	**0.76 (0.59–0.97)**	**0.030**

SNP, single nucleotide polymorphism; OR, odds ratio; 95% CI, 95% confidence interval.

p values were calculated by logistic regression analysis with adjustments for age and gender.

p < 0.05 means the data is statistically significant. Bold indicates that the values have statistical significance.

We further conducted subgroup analyses by age to examine the effects of LRRC3B SNPs on BC risk (**Table 3**). LRRC3B rs1907168 was related to the reduced risk of BC among young participants (age < 51 years) under the allele (OR = 0.69, 95% CI: 0.48–0.99, *p* = 0.043), heterozygote (OR = 0.53, 95% CI: 0.35–0.80, *p* = 0.002), dominant (OR = 0.58, 95% CI: 0.39–0.86, *p* = 0.008) and log-additive (OR = 0.68, 95% CI: 0.47–0.98, *p* = 0.038) models. The significance of the heterozygote model still existed after Bonferroni correction. In addition, rs78205284 polymorphism (TT *vs*. GG, OR = 2.83, 95% CI: 1.08–7.41, *p* = 0.034; and TT *vs*. GG-GT, OR = 2.72, 95% CI: 1.05–7.07, *p* = 0.040) contributed to the increased BC risk at age ≤51 years.

**Table 3:** Relationships between *LRRC3B* polymorphisms and breast cancer risk according to the stratification by age.

SNP ID	Model	Genotype	> 51				≤51			
			Case	Control	OR (95%CI)	*p*	Case	Control	OR (95%CI)	*p*
rs1907168	Allele	A	515	500	1.00	0.336	472	429	1.00	**0.043**
		T	70	90	0.85 (0.62–1.18)		60	79	**0.69 (0.48**–**0.99)**	
	Genotype	AA	220	211	1.00		212	177	1.00	
		AT	75	78	0.91 (0.63–1.32)	0.609	48	75	**0.53 (0.35**–**0.80)**	0.002*
		TT	2	6	0.32 (0.06–1.59)	0.163	6	2	2.54 (0.51–12.77)	0.257
	Dominant	AA	220	211	1.00	0.437	212	177	1.00	**0.008**
		AT-TT	77	84	0.87 (0.60–1.25)		54	77	**0.58 (0.39**–**0.86)**	
	Recessive	AA-AT	295	289	1.00	0.172	260	252	1.00	0.188
		TT	2	6	0.33 (0.07–1.63)		6	2	2.95 (0.59–14.77)	
	Log-additive	---	---	---	0.83 (0.59–1.17)	0.287	---	---	**0.68 (0.47**–**0.98)**	**0.038**
rs78205284	Allele	G	482	477	1.00	0.993	419	428	1.00	0.063
		T	110	109	1.00 (0.74–1.34)		113	86	1.34 (0.98–1.83)	
	Genotype	GG	201	197	1.00		169	177	1.00	
		GT	80	83	0.95 (0.66–1.37)	0.790	81	74	1.14 (0.78–1.66)	0.505
		TT	15	13	1.15 (0.53–2.48)	0.725	16	6	**2.83 (1.08**–**7.41)**	**0.034**
	Dominant	GG	201	197	1.00	0.900	169	177	1.00	0.207
		GT-TT	95	96	0.98 (0.69–1.38)		97	80	1.26 (0.88–1.82)	
	Recessive	GG-GT	281	280	1.00	0.694	250	251	1.00	**0.040**
		TT	15	13	1.17 (0.54–2,50)		16	6	**2.72 (1.05**–**7.07)**	
	Log-additive	---	---	---	1.01 (0.76–1.33)	0.966			1.33 (0.98-1.80)	0.071

SNP, single nucleotide polymorphism; OR, odds ratio; 95% CI, 95% confidence interval.

p values were calculated by logistic regression analysis with adjustments for age.

p < 0.05 indicates statistical significance. Bold indicates that the values have statistical significance.

*p indicates that after Bonferroni correction (p < 0.05/10) means the data is statistically significant.

We also investigated the potential associations of *LRRC3B* polymorphisms with clinicopathological characteristics of BC, including tumor size, lymph node metastasis, clinical stage, the status of ER, PR, Her-2, and Ki67 (**Table 4**). We found that rs6551122 (OR = 0.51, *p* = 0.028) and rs12635768 (homozygote, OR = 0.36, *p* = 0.023; recessive, OR = 0.39, *p* = 0.032; log-additive, OR = 0.69, *p* = 0.043) polymorphisms were related to a smaller BC tumor size (<2 cm). In addition, rs112276562, rs6551122, and rs73150416 variants contributed to a lower incidence of PR-positive BC (for rs112276562, heterozygote OR = 0.56, *p* = 0.002 and dominant OR = 0.63, *p* = 0.011; for rs6551122, heterozygote OR = 0.63, *p* = 0.016 and dominant OR = 0.64, *p* = 0.018; and for rs73150416, heterozygote OR = 0.57, *p* = 0.005, dominant OR = 0.61, *p* = 0.008, log-additive OR = 0.72, *p* = 0.035, and allele OR = 0.71, *p* = 0.029). After Bonferroni correction, rs112276562 was associated with a lower incidence of PR-positive BC under the heterozygote model. Moreover, rs6788033 was associated with a lower the expression level of Ki-67 in the dominant (OR = 0.64, *p* = 0.030), log-additive (OR = 0.68, *p* = 0.024) and allele (OR = 0.69, *p* = 0.025) models. There was no significant association of *LRRC3B* polymorphisms with respect to the other variables like lymph node metastasis, clinical stage, and ER/HER2 status.

**Table 4:** The association between *LRRC3B* polymorphisms and clinical features of breast cancer.

SNPs ID	Variables		OR (95%), *p*					
			Homozygote	Heterozygote	Dominant	Recessive	Log-additive	Allele
rs112276562	Tumor size	<2 cm/≥2 cm	0.90 (0.31–2.64), 0.847	1.39 (0.83–2.34), 0.212	1.31 (0.80–2.13), 0.281	0.82 (0.28–2.40), 0.721	1.17 (0.78–1.76), 0.441	1.16 (0.77–1.75), 0.486
	LNM	(−)/(+)	0.61 (0.25–1.47), 0.272	0.81 (0.56–1.18), 0.271	0.78 (0.55–1.12), 0.183	0.65 (0.27–1.56), 0.337	0.80 (0.59–1.08), 0.145	0.80 (0.59–1.08), 0.145
	Stage	I–II/III–IV	0.75 (0.29–1.96), 0.560	0.81 (0.54–1.23), 0.326	0.80 (0.54–1.20), 0.282	0.80 (0.31–2.07), 0.648	0.83 (0.60–1.17), 0.289	0.84 (0.60–1.18), 0.310
	ER	(−)/(+)	1.78 (0.59–5.40), 0.308	0.71 (0.48–1.06), 0.090	0.78 (0.53–1.14), 0.200	2.00 (0.66–6.00), 0.218	0.90 (0.65–1.24), 0.524	0.90 (0.65–1.24), 0.517
	PR	(−)/(+)	1.54 (0.59–4.03), 0.375	**0.56 (0.39**–0.81), 0.002*	**0.63 (0.44**–**0.90), 0.011**	1.87 (0.72–4.84), 0.196	0.77 (0.57–1.04), 0.093	0.77 (0.57–1.04), 0.085
	HER2	(−)/(+)	0.88 (0.27–2.91), 0.835	1.30 (0.76–2.22), 0.339	1.24 (0.74–2.06), 0.410	0.81 (0.25–2.66), 0.732	1.14 (0.74–1.76), 0.561	1.11 (0.72–1.72), 0.626
	Ki-67	<10%/≥10%	1.17 (0.41–3.34), 0.768	1.06 (0.69–1.64), 0.790	1.07 (0.70–1.63), 0.743	1.15 (0.41–3.24), 0.794	1.07 (0.75–1.53), 0.713	1.06 (0.74–1.51), 0.761
rs6551122	Tumor size	<2 cm/≥2 cm	0.59 (0.30–1.13), 0.109	1.32 (0.81–2.14), 0.268	1.08 (0.69–1.69), 0.734	**0.51 (0.28**–**0.93), 0.028**	0.87 (0.63–1.20), 0.393	0.88 (0.64–1.21), 0.420
	LNM	(−)/ (+)	0.76 (0.44–1.30), 0.312	0.92 (0.64–1.33), 0.670	0.88 (0.62–1.26), 0.492	0.79 (0.48–1.31), 0.359	0.88 (0.69–1.14), 0.337	0.89 (0.70–1.14), 0.367
	Stage	I–II/III–IV	0.66 (0.36v1.23), 0.194	1.06 (0.71–1.57), 0.794	0.96 (0.65–1.40), 0.825	0.64 (0.36–1.15), 0.135	0.88 (0.67–1.16), 0.355	0.89 (0.68–1.16), 0.389
	ER	(−)/(+)	1.05 (0.58–1.92), 0.866	0.84 (0.56–1.25), 0.380	0.88 (0.60–1.29), 0.504	1.17 (0.67–2.03), 0.582	0.97 (0.74–1.28), 0.842	0.98 (0.75–1.28), 0.866
	PR	(−)/(+)	0.71 (0.41–1.23), 0.218	**0.63 (0.43**–**0.92), 0.016**	**0.64 (0.45**–**0.93), 0.018**	0.93 (0.57–1.53), 0.776	0.79 (0.61–1.02), 0.064	0.80 (0.62–1.03), 0.078
	HER2	(−)/(+)	0.96 (0.42–2.17), 0.918	0.83 (0.49–1.39), 0.475	0.85 (0.52–1.40), 0.524	1.07 (0.50–2.28), 0.860	0.93 (0.65-1.34), 0.699	0.93 (0.66–1.32), 0.690
	Ki-67	<10%/≥10%	1.32 (0.69–2.52), 0.408	1.38 (0.90–2.13), 0.140	1.37 (0.91-2.06), 0.133	1.10 (0.60–2.02), 0.757	1.21 (0.90–1.65), 0.212	1.21 (0.90–1.63), 0.203
rs73150416	Tumor size	<2 cm/≥2 cm	0.71 (0.26–1.95), 0.505	1.22 (0.71–2.07), 0.473	1.11 (0.68-1.82), 0.676	0.67 (0.25–1.85), 0.444	1.01 (0.68–1.51), 0.953	0.99 (0.66–1.50), 0.972
	LNM	(−)/ (+)	0.54 (0.22–1.34), 0.183	0.79 (0.54–1.16), 0.226	0.75 (0.52–1.09), 0.130	0.58 (0.24–1.42), 0.232	0.77 (0.56–1.04), 0.091	0.76 (0.55–1.04), 0.087
	Stage	I–II/III–IV	0.47 (0.16–1.43), 0.183	0.84 (0.55–1.28), 0.405	0.78 (0.52–1.18), 0.236	0.50 (0.16–1.49), 0.211	0.78 (0.55–1.10), 0.151	0.78 (0.54–1.11), 0.159
	ER	(−)/ (+)	1.29 (0.46–3.60), 0.634	0.75 (0.50–1.13), 0.169	0.80 (0.54–1.18), 0.261	1.40 (0.50–3.89), 0.519	0.89 (0.64–1.23), 0.474	0.88 (0.63–1.24), 0.464
	PR	(−)/(+)	0.95 (0.39–2.34), 0.916	**0.57 (0.39**–**0.84), 0.005**	**0.61 (0.42**–**0.88), 0.008**	1.13 (0.46–2.74), 0.792	**0.72 (0.53**–**0.98), 0.035**	**0.71 (0.52**–**0.97), 0.029**
	HER2	(−)/(+)	1.26 (0.34–4.66), 0.730	1.11 (0.65–1.89), 0.704	1.13 (0.67–1.88), 0.652	1.22 (0.33–4.48), 0.764	1.11 (0.72–1.72), 0.628	1.09 (0.70–1.69), 0.700
	Ki-67	<10%/≥10%	1.01 (0.38–2.67), 0.987	1.03 (0.66–1.62), 0.895	1.03 (0.67–1.58), 0.900	1.00 (0.38–2.62), 0.999	1.02 (0.72–1.45), 0.918	1.01 (0.70–1.45), 0.966
rs12635768	Tumor size	<2 cm/≥2 cm	**0.36 (0.15**–**0.87), 0.023**	0.81 (0.50–1.30), 0.378	0.72 (0.46–1.12), 0.144	**0.39 (0.16**–**0.92), 0.032**	**0.69 (0.48-0.99), 0.043**	0.71 (0.50–1.01), 0.058
	LNM	(−)/(+)	1.27 (0.59–2.71), 0.541	1.11 (0.77–1.59), 0.577	1.13 (0.8–1.59), 0.495	1.22 (0.58–2.57), 0.605	1.12 (0.84–1.48), 0.448	1.12 (0.84–1.48), 0.434
	Stage	I–II/III–IV	1.32 (0.58–2.98), 0.506	1.16 (0.79–1.72), 0.454	1.18 (0.81–1.72), 0.386	1.24 (0.56–2.76), 0.594	1.16 (0.85–1.57), 0.359	1.15 (0.85–1.56), 0.364
	ER	(−)/(+)	0.88 (0.38–2.01), 0.759	0.92 (0.62–1.35), 0.660	0.91 (0.63–1.33), 0.627	0.91 (0.40–2.05), 0.818	0.93 (0.68–1.26), 0.625	0.93 (0.69–1.27), 0.658
	PR	(−)/(+)	0.96 (0.44–2.09), 0.910	0.87 (0.60–1.25), 0.448	0.88 (0.62–1.25), 0.470	1.01 (0.47–2.18), 0.981	0.92 (0.69–1.22), 0.557	0.93 (0.70–1.24), 0.628
	HER2	(−)/(+)	1.11 (0.35–3.51), 0.857	0.71 (0.43–1.16), 0.173	0.75 (0.46–1.21), 0.233	1.27 (0.41–3.95), 0.675	0.85 (0.57–1.26), 0.407	0.86 (0.58–1.27), 0.447
	Ki-67	<10%/≥10%	1.80 (0.59–5.44), 0.300	1.06 (0.69–1.62), 0.786	1.12 (0.74–1.69), 0.589	1.76 (0.59–5.26), 0.314	1.16 (0.82–1.64), 0.411	1.18 (0.84–1.67), 0.345
rs6788033	Tumor size	<2 cm/≥2 cm	1.12 (0.43–2.96), 0.813	1.29 (0.81–2.07), 0.282	1.27 (0.81–1.99), 0.297	1.02 (0.39–2.64), 0.973	1.18 (0.81–1.72), 0.384	1.19 (0.82–1.72), 0.360
	LNM	(−)/(+)	0.56 (0.24–1.32), 0.183	1.02 (0.71–1.45), 0.924	0.95 (0.68–1.35), 0.791	0.56 (0.24–1.29), 0.172	0.90 (0.67–1.20), 0.474	0.90 (0.68–1.20), 0.473
	Stage	I–II/III–IV	0.33 (0.10–1.14), 0.079	1.02 (0.69–1.50), 0.941	0.92 (0.63–1.35), 0.685	0.33 (0.10–1.12), 0.076	0.85 (0.61–1.17), 0.317	0.85 (0.62–1.16), 0.302
	ER	(−)/ (+)	1.39 (0.54–3.58), 0.500	1.02 (0.69–1.50), 0.933	1.05 (0.72–1.53), 0.795	1.38 (0.54–3.52), 0.503	1.08 (0.78–1.48), 0.645	1.07 (0.78–1.47), 0.660
	PR	(−)/(+)	1.72 (0.70–4.26), 0.239	1.11 (0.77–1.60), 0.572	1.16 (0.82–1.65), 0.401	1.66 (0.68–4.05), 0.269	1.19 (0.88–1.60), 0.268	1.17 (0.88–1.57), 0.286
	HER2	(−)/(+)	0.52 (0.17–1.62), 0.259	0.78 (0.47–1.28), 0.325	0.75 (0.46–1.21), 0.234	0.57 (0.19–1.76), 0.330	0.75 (0.50–1.13), 0.175	0.78 (0.53–1.16), 0.215
	Ki-67	<10%/≥10%	0.50 (0.21–1.20), 0.119	0.66 (0.44–1.01), 0.054	**0.64 (0.43**–**0.96), 0.030**	0.59 (0.25–1.39), 0.225	**0.68 (0.49**–**0.95), 0.024**	**0.69 (0.50**–**0.95), 0.025**

SNP, single nucleotide polymorphism; OR, odds ratio; 95% CI, 95% confidence interval; ER, estrogen receptor; RP, progesterone receptor; HER2, human epidermal growth factor receptor 2; LNM, lymph node metastasis.

p values were calculated by logistic regression analysis with adjustments for age.

p < 0.05 indicates statistical significance.

*p indicates that after Bonferroni correction (p < 0.05/10) means the data is statistically significant. Bold indicates that the values have statistical significance.

FPRP analysis was carried out to interrogate whether the significant findings were deserving attention (**Table 5**). At the prior probability level of 0.1, the significant association for rs1907168 (heterozygote model, FPRP = 0.184) remained noteworthy in the overall analysis. In the subgroup at age <51 years, significant findings remained noteworthy for rs1907168 under the heterozygote (FPRP = 0.141) and dominant (FPRP = 0.198) models. Moreover, the associations of rs112276562 (heterozygote model, FPRP = 0.095) and rs73150416 (heterozygote model, FPRP = 0.159; and dominant model FPRP = 0.189) with PR-positive BC were also positive at the prior probability level of 0.1.

**Table 5:** False-positive report probability values for the associations between *LRRC3B* Polymorphisms and BC susceptibility.

Group/ SNPs ID	Model	OR (95% CI)	*p*	Statistical power	Prior probability				
					0.25	0.1	0.01	0.001	0.0001
**Overall**									
rs1907168	Allele	0.77 (0.61–0.99)	0.037	0.869	**0.125**	0.301	0.825	0.979	0.998
	Heterozygote	0.71 (0.54–0.94)	0.017	0.670	**0.070**	**0.184**	0.712	0.961	0.996
	Dominant	0.72 (0.55–0.95)	0.019	0.707	**0.079**	0.205	0.739	0.996	0.997
	Log-additive	0.76 (0.59–0.97)	0.030	0.854	**0.088**	0.225	0.761	0.970	0.997
**Age < 51 years**									
rs1907168	Allele	0.69 (0.48–0.99)	0.043	0.574	**0.187**	0.408	0.883	0.987	0.999
	Heterozygote	0.53 (0.35–0.80)	0.002	0.609	**0.052**	**0.141**	0.644	0.948	0.995
	Dominant	0.58 (0.39–0.86)	0.008	0.770	**0.076**	**0.198**	0.731	0.965	0.996
	Log-additive	0.68 (0.47–0.98)	0.038	0.542	**0.176**	0.391	0.876	0.986	0.999
rs78205284	Homozygote	2.83 (1.08–7.41)	0.034	0.240	0.299	0.562	0.934	0.993	0.999
	Recessive	2.72 (1.05–7.07)	0.040	0.264	0.313	0.577	0.938	0.993	0.999
**PR**									
rs112276562	Heterozygote	0.56 (0.39–0.81)	0.002	0.726	**0.034**	**0.095**	0.537	0.921	0.992
	Dominant	0.63 (0.44–0.90)	0.011	0.898	**0.081**	0.209	0.744	0.967	0.997
rs6551122	Heterozygote	0.63 (0.43–0.92)	0.016	0.884	**0.116**	0.282	0.812	0.978	0.998
	Dominant	0.64 (0.45–0.93)	0.018	0.902	**0.122**	0.294	0.821	0.979	0.998
rs73150416	Heterozygote	0.57 (0.39–0.84)	0.005	0.746	**0.059**	**0.159**	0.675	0.954	0.995
	Dominant	0.61 (0.42–0.88)	0.008	0.856	**0.072**	**0.189**	0.719	0.963	0.996
	Log-additive	0.72 (0.53–0.98)	0.035	0.688	**0.138**	0.325	0.841	0.982	0.998
	Allele	0.71 (0.52–0.97)	0.029	0.654	**0.126**	0.302	0.826	0.980	0.998
**Tumor size**									
rs6551122	Recessive	0.51 (0.28–0.93)	0.028	0.526	**0.138**	0.324	0.841	0.892	0.998
rs12635768	Recessive	0.39 (0.16–0.92)	0.032	0.285	0.249	0.499	0.916	0.991	0.999
	Log-additive	0.69 (0.48–0.99)	0.043	0.574	**0.187**	0.408	0.883	0.987	0.999
**Ki-67**									
rs6788033	Dominant	0.64 (0.43–0.96)	0.030	0.884	**0.181**	0.398	0.879	0.987	0.999
	Log-additive	0.68 (0.49–0.95)	0.024	0.546	**0.116**	0.282	0.812	0.978	0.998
	Allele	0.69 (0.50–0.95)	0.025	0.584	**0.106**	0.261	0.796	0.975	0.997

SNP, single nucleotide polymorphism; OR, odds ratio; 95% CI, 95% confidence interval; RP, progesterone receptor.

p values were calculated by logistic regression analysis with adjustments for age.

Statistical power was calculated using the number of observations in the subgroup and the OR and p values in this table.

The level of false-positive report probability threshold was set at 0.2, and noteworthy findings are presented. Bold indicates that the values have statistical significance.

**Figure 1:**
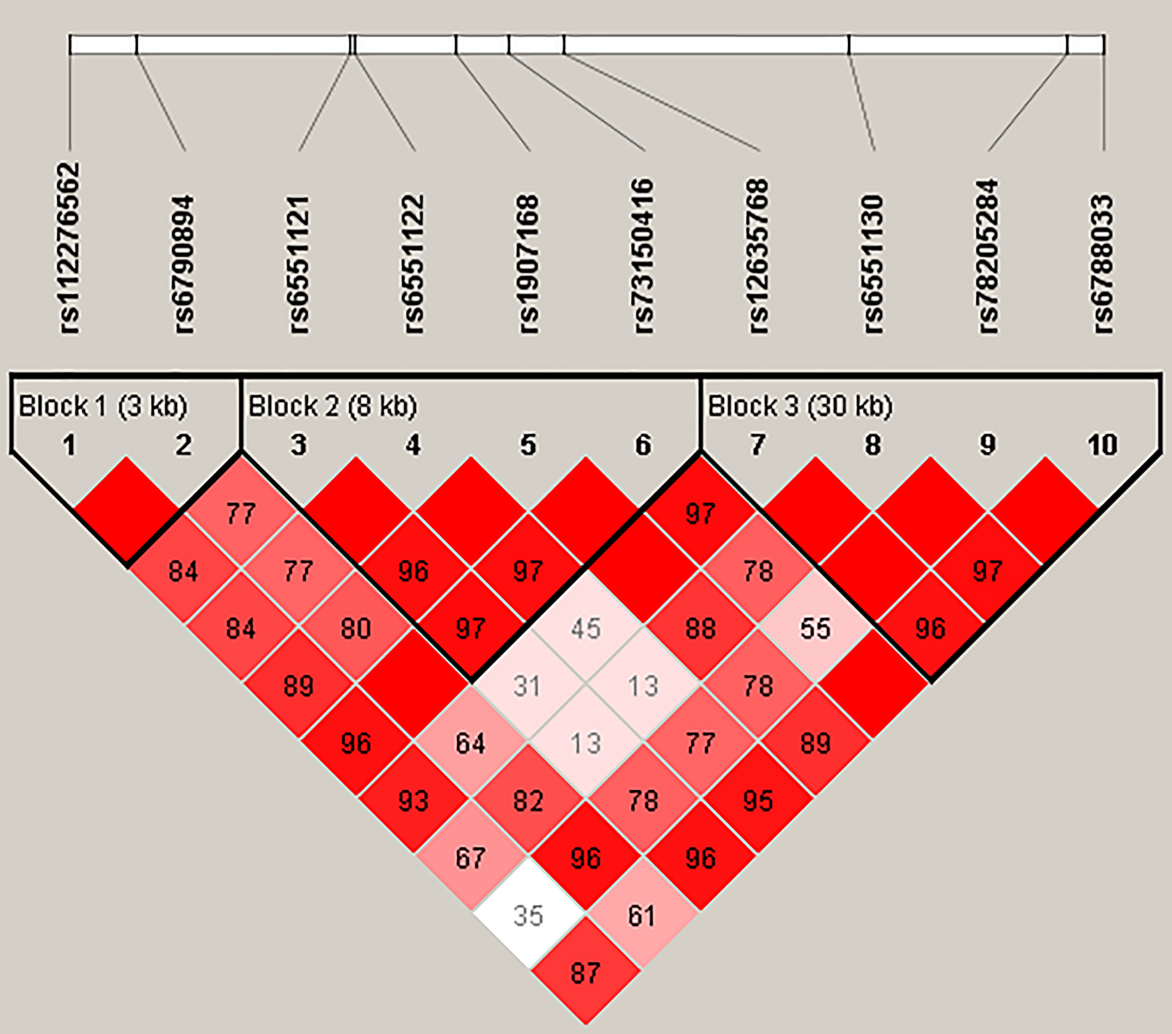
Haplotype block map for ten SNPs in the *LRRC3B* gene. The numbers inside the diamonds indicate the D′ for pairwise analyses.

**Figure 1** represented the reconstructed LD plot and had three blocks in *LRRC3B* SNPs (Block 1, rs112276562 and rs6790894; Block 2, rs6551121, rs6551122, rs1907168, and rs73150416; and Block 3, rs12635768, rs6551130, rs78205284, and rs6788033). Moreover, ‘GATT’ haplotype in Block 2 had a higher risk for BC (OR = 1.29, 95% CI: 1.00–1.65, *p* = 0.048, **Supplementary Table 4**).

**Figure 2:**
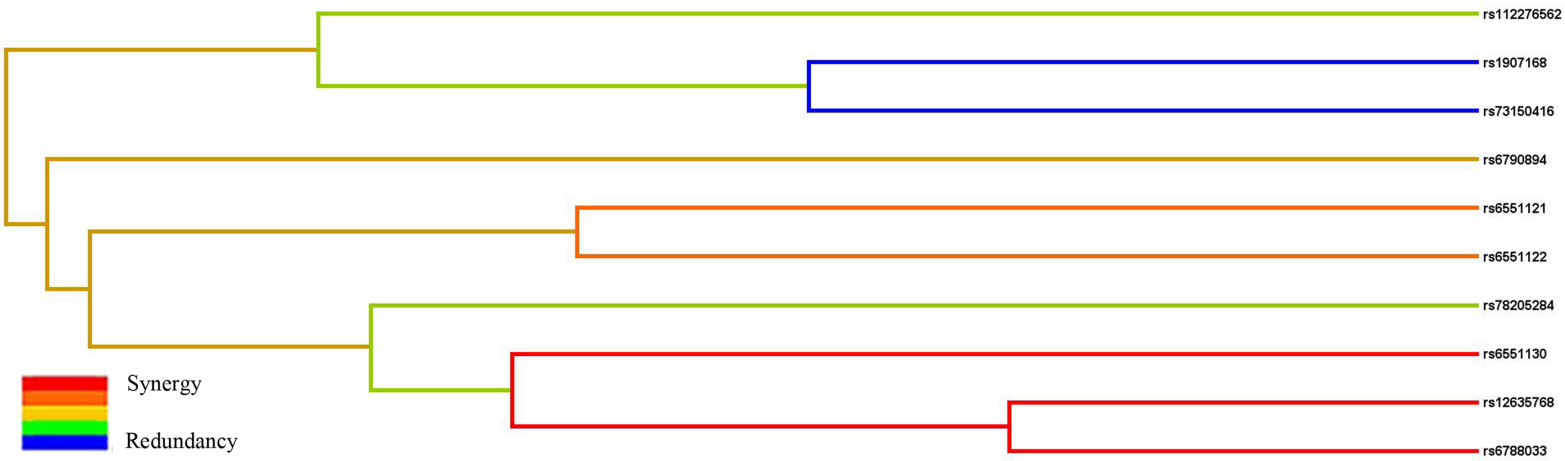
The interaction dendrogram derived from MDR for the SNP–SNP interaction in *LRRC3B*. Short connections among nodes represent stronger synergistic (red and orange) or redundant (green and blue) interactions.

Subsequently, MDR analysis was implemented to assess the impact of the SNP–SNP interactions. Dendrogram interaction analysis of our data showed that rs6551130, rs12635768, and rs6788033 exhibited a strong synergy effect, and rs1907168 and rs73150416 had a strong redundancy effect, as shown in **Figure 2**. **Table 6** showed that rs1907168 was the best single-locus model to predict the risk of BC (testing accuracy = 0.5048, CVC = 8/10, *p* = 0.0298). The best multi-loci model included rs112276562, rs1907168, and rs6551130 (testing accuracy = 0.5205, CVC = 7/10, *p <*0.0001).

**Table 6:** SNP–SNP interaction models of *LRRC3B* gene analyzed by the MDR method.

Model	Training Bal. Acc.	Testing Bal. Acc.	CVC	*p*
rs1907168	0.5309	0.5048	8/10	**0.0298**
rs6551130, rs6788033	0.5469	0.4966	5/10	**0.0030**
rs112276562, rs1907168, rs6551130	0.5636	0.5205	7/10	**<0.0001**
rs112276562, rs12635768, rs1907168, rs6551130	0.5783	0.5083	3/10	**<0.0001**
rs112276562, rs12635768, rs6551121, rs6551130, rs6790894	0.5941	0.4933	6/10	**<0.0001**
rs112276562, rs12635768, rs6551121, rs6551122, rs6551130, rs6790894	0.6105	0.5085	4/10	**<0.0001**
rs112276562, rs12635768, rs1907168, rs6551121, rs6788033, rs6790894, rs78205284	0.6212	0.5021	9/10	**<0.0001**
rs112276562, rs12635768, rs1907168, rs6551121, rs6551130, rs6788033, rs6790894, rs78205284	0.6275	0.5029	9/10	**<0.0001**
rs112276562, rs12635768, rs1907168, rs6551121, rs6551122, rs6551130, rs6788033, rs6790894, rs78205284	0.6284	0.5172	9/10	**<0.0001**
rs112276562, rs12635768, rs1907168, rs6551121, rs6551122, rs6551130, rs6788033, rs6790894, rs73150416, rs78205284	0.628	0.5163	10/10	**<0.0001**

MDR, multifactor dimensionality reduction; Bal. Acc., balanced accuracy; CVC, cross-validation consistency; OR, odds ratio; CI, confidence interval.

p values were calculated using χ^2^ tests.

Bold indicates that p < 0.05 indicates statistical significance.

## Discussion

This study investigated the effect of *LRRC3B* genetic variation on BC risk in a Chinese Han population. The result revealed that rs1907168 conferred to the reduced BC risk overall. In additional, rs1907168 decreased the risk of BC while rs78205284 increased BC susceptibility among the population at age ≤51 years. Noticeably, clinical parameters such as the status of PR and Ki67 and tumor size were significantly associated with *LRRC3B* polymorphism. Furthermore, we found the existence of three blocks in *LRRC3B* SNPs and the association of ‘GATT’ haplotype with an increased BC risk. Our results suggested *LRRC3B* variants might be susceptibility markers for BC. Specially; this is firstly demonstrated by the effects of *LRRC3B* SNPs on BC susceptibility.

*LRRC3B*, located at 3p24, contained a leucine-rich repeat N-terminal domain (Leu >13%). The protein-conserved region of LRRC3B exists at cAMP- and cGMP-dependent protein kinase phosphorylation sites, thus, related to cell differentiation, cycle regulation, proliferation and invasion (20, 21). Several studies have revealed that *LRRC3B* could be a tumor suppressor in carcinogenesis (22, 23). Formerly, the expression of *LRRC3B* was reduced in BC tissues and associated with tumor grade of BC (13). Consistently, we also found that *LRRC3B* was down-regulated in BC, and low expression of *LRRC3B* was related to a poor overall survival for BC based on bioinformatics analysis. Moreover, *LRRC3B* might serve a protective role in preventing bupivacaine-induced BC recurrence and metastasis (15). These results hint us that *LRRC3B* has an important role in BC occurrence and progression.

Genetic variations in *LRRC3B* might be associated with the development and progression of disease. However, there is no finding of related research on *LRRC3B* polymorphisms. In this study, *LRRC3B* rs1907168 (A/T) variant had a protective effect in BC risk. Further, stratified analysis showed that rs1907168 decreased while rs78205284 increased BC susceptibility among the population at age ≤51 years. Especially, rs1907168 was associated with the decreased BC risk after Bonferroni correction. Notably, the peak age for BC is 40–50 years in the Asian countries, whereas 60–70 years in the Western countries (24). This finding suggested that age might affect the association of genetic variations and BC risk, and that rs78205284 was a risk factor to BC occurrence among younger women in the Chinese Han population.

As BC is a complex disease, the same variant might show a different response for BC risk across different tumor sizes, clinical stages and clinical features (25, 26). We further evaluated the association of *LRRC3B* polymorphisms with clinical features of BC, including tumor size, lymph node metastasis, clinical stage, the status of ER, PR, Her-2, and Ki67. We found that rs6551122 and rs12635768 polymorphisms were associated with a smaller BC tumor size (<2 cm). In addition, rs112276562, rs6551122, and rs73150416 variants contributed to a lower incidence of PR-positive BC. Especially, rs112276562 was associated with a lower incidence of PR-positive BC after Bonferroni correction. Our study suggested that *LRRC3B* polymorphisms might be involved in the pathogenesis of PR-positive BC. Moreover, rs6788033 was associated with a lower level of Ki-67. Our findings showed *LRRC3B* polymorphisms were associated with tumor size and PR/Ki-67 status, which might act as predictors for BC occurrence and development.

Our study provided evidence that *LRRC3B* variants were associated with BC susceptibility and related to clinical characteristic of BC patients. Inevitably, there were several intrinsic limitations in this study. First, data on *LRRC3B* SNPs’ potential function and *LRRC3B* expression/survival in BC were predicted in *in silico* analysis only; thus, further functional assay is necessary to explore the functions and the underlying mechanisms of these polymorphisms. Second, gene-to-environment interactions could not be further analyzed due to the unavailability of relevant information; therefore, additional studies will be required.

## Conclusion

In conclusion, we firstly reported that *LRRC3B* polymorphisms might contribute to individual susceptibility and progression to BC among Chinese Han females. The study might help in understanding the possible effect of *LRRC3B* variants in the development of BC.

## Data Availability Statement

The datasets presented in this study can be found in online repositories. The names of the repository/repositories and accession number(s) can be found in the article/**Supplementary Material**.

## Ethics Statement

The studies involving human participants were reviewed and approved by Nanchang University. The patients/participants provided their written informed consent to participate in this study.

## Author Contributions

The author confirms being the sole contributor of this work and has approved it for publication.

## Conflict of Interest

The author declares that the research was conducted in the absence of any commercial or financial relationships that could be construed as a potential conflict of interest.

## Supplementary Material

Supplementary Figure 1
*LRRC3B* gene expression is down-regulated in BC compared with that in normal lung tissues. Each bar represents the average level of *LRRC3B* expression. Error bars represent the standard deviation of the mean value. The data were extracted from the GEPIA database (http://gepia.cancer-pku.cn/). * indicates statistical significance (p < 0.01).

Supplementary Figure 2
*LRRC3B* low expression is associated with poor survival in BC. Kaplan–Meier plots of overall survival: comparison of patients with high *vs*. low expression of *LRRC3B* in BC patients. The Kaplan–Meier plots were generated by the Kaplan–Meier Plotter (http://kmplot.com/analysis/).
