# Supplementary figures and images for: LRRC3B Polymorphisms Contributed to Breast Cancer Susceptibility in Chinese Han Population
Source: Front Oncol. 2021 Jun 10;11:657168. doi: 10.3389/fonc.2021.657168 (PMC8222685; doi:10.3389/fonc.2021.657168)

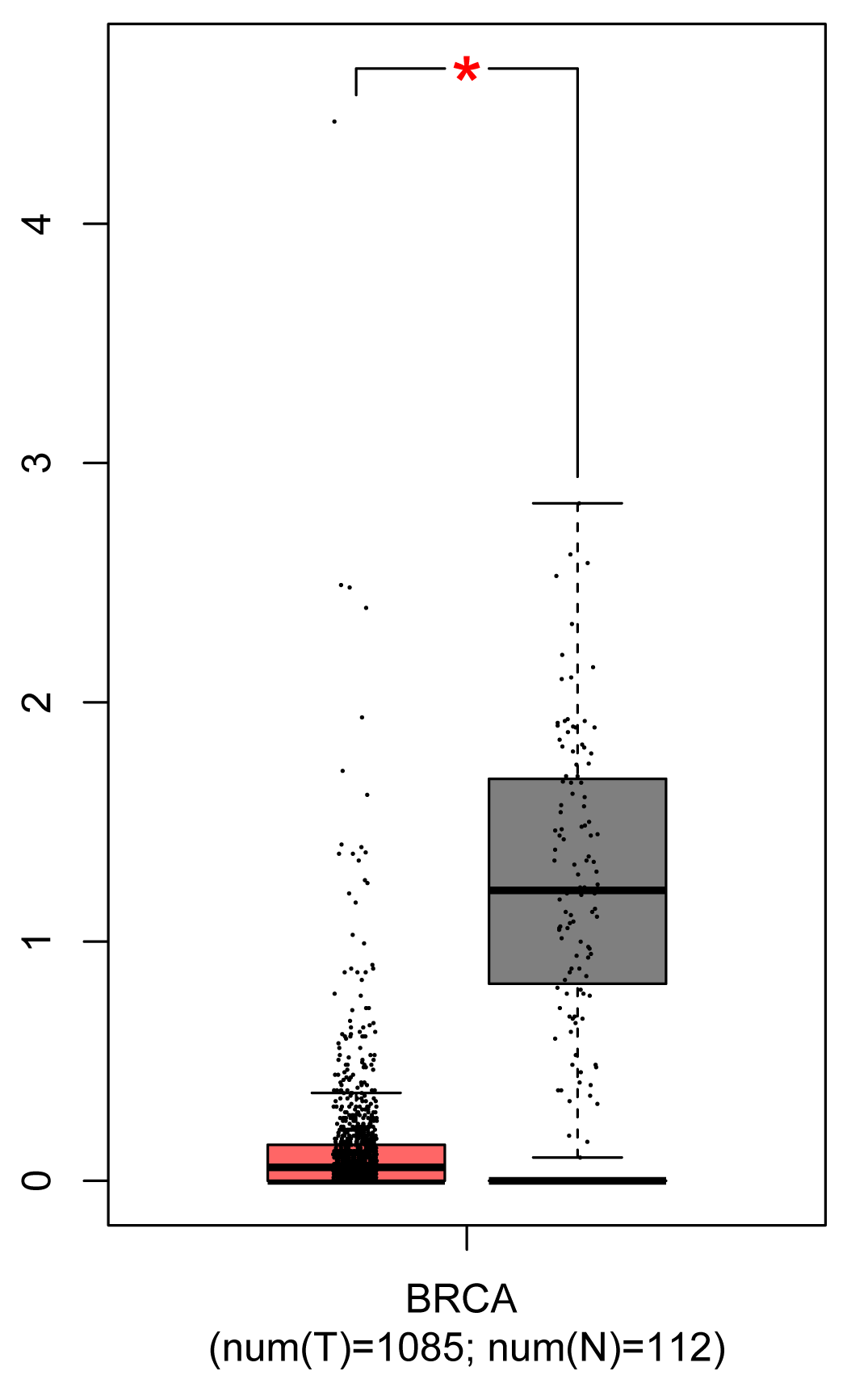

Supplement: Supplementary Figure 1 — LRRC3B gene expression is down-regulated in BC compared with that in normal lung tissues. Each bar represents the average level of LRRC3B expression. Error bars represent the standard deviation of the mean value. The data were extracted from the GEPIA database (http://gepia.cancer-pku.cn/). * indicates statistical significance (p < 0.01). [file Image_1.tif]

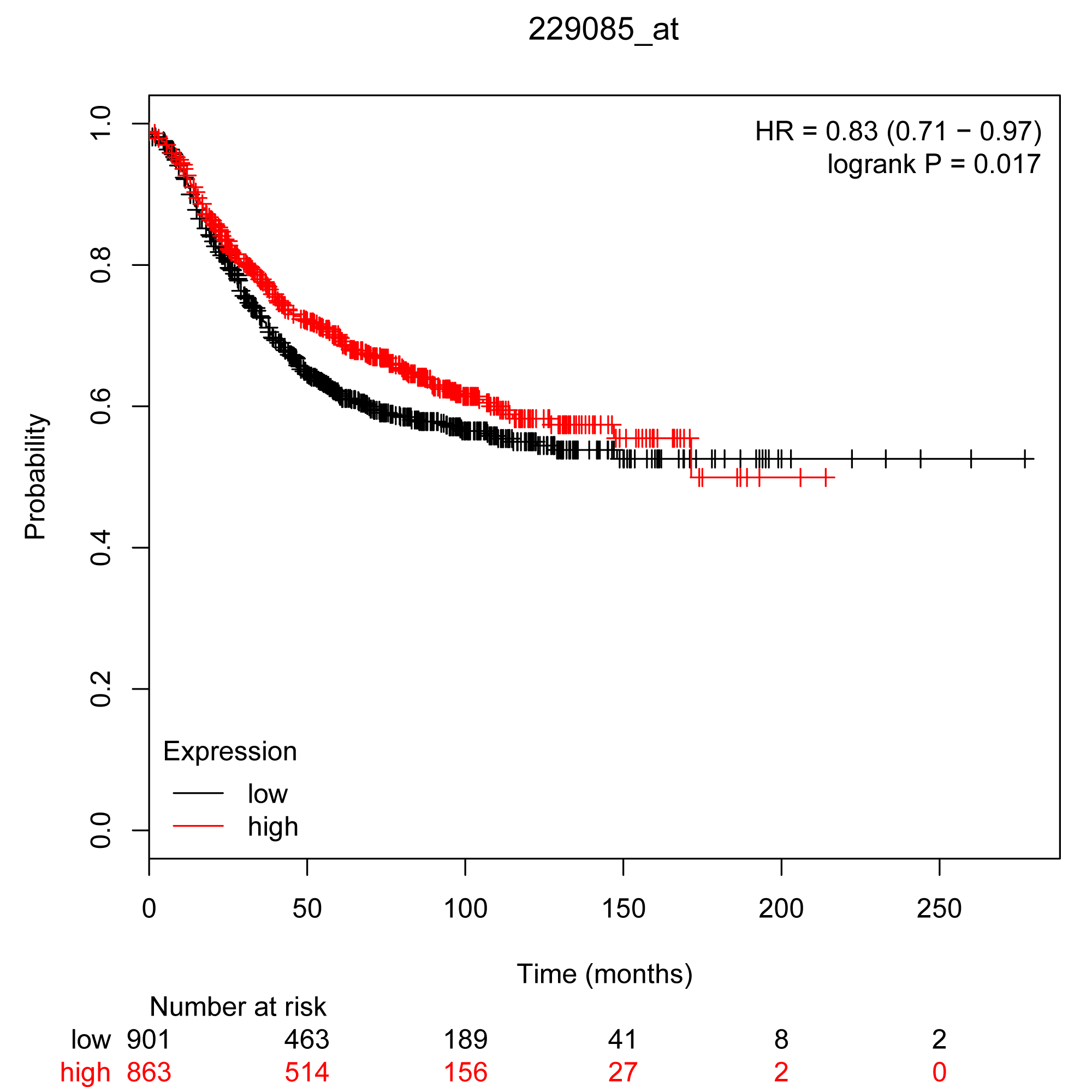

Supplement: Supplementary Figure 2 — LRRC3B low expression is associated with poor survival in BC. Kaplan–Meier plots of overall survival: comparison of patients with high vs. low expression of LRRC3B in BC patients. The Kaplan–Meier plots were generated by the Kaplan–Meier Plotter (http://kmplot.com/analysis/). [file Image_2.tif]
