# Supplementary material for: LRRC3B Polymorphisms Contributed to Breast Cancer Susceptibility in Chinese Han Population
Source: Front Oncol. 2021 Jun 10;11:657168. doi: 10.3389/fonc.2021.657168 (PMC8222685; doi:10.3389/fonc.2021.657168)
Supplement: Supplementary file 3 [file DataSheet_1.docx]

Supplementary **Table 1. Primers sequence of PCR and UEP used in this study**

| **SNP** | **First Primer(5'-3')** | **Second Primer (5'-3')** | **UEP SEQ (5'-3')** |
| --- | --- | --- | --- |
| rs112276562 | ACGTTGGATGAATCACAGCTATTCTGGAGG | ACGTTGGATGCTTTTGAGACGGAGTCTAA | ACGGAGTCTAACTGTCA |
| rs6790894 | ACGTTGGATGCTGAGTTGATCCAGGAAAGG | ACGTTGGATGTATGAAGTCTCCAGGTGTCC | tttctCTGTCTGGGAGAACACC |
| rs6551121 | ACGTTGGATGGTTTTAGAGATTTCAAGTC | ACGTTGGATGTGGGGAGATGCTTTGTTTAC | AGTGCTGTTATAACTGTTAGAATTTTA |
| rs6551122 | ACGTTGGATGCTCATATCTCTTACATGCTG | ACGTTGGATGTGCTTGTTGACCCAGCAATG | ggACTGGTGGAAATGACAG |
| rs1907168 | ACGTTGGATGGAACAGATCACACTTTCCCC | ACGTTGGATGAGAGTAATAACCAGGCTGCG | GGCTGCGCCTGTGAAAAT |
| rs73150416 | ACGTTGGATGAATGGCTACATAACATCAC | ACGTTGGATGGAATGGGGAAGACATAAGTG | TTGGCTAGAGTAACTATAATATATCC |
| rs12635768 | ACGTTGGATGTTCTGTGGTGGAGTTCTAGG | ACGTTGGATGCCAATGCTGATAGATGCGAG | tcctaACCCCTTGTGGGAACAAATC |
| rs6551130 | ACGTTGGATGGGCATATTTTATGCACAGGC | ACGTTGGATGGCGTGCAATTCCTACTCTAC | GTCCAAACAAACAATTGAC |
| rs78205284 | ACGTTGGATGCCAAGCTACTCACGGATTTG | ACGTTGGATGGGTAAAATAGCCATGTGTATC | GCCATGTGTATCAGTTAGCTTTC |
| rs6788033 | ACGTTGGATGGTAAGAAGAGGCACTATGTC | ACGTTGGATGGGAGGCATTGATAGACAACC | cccaCTATTCCGTGTTAAGTGCTAT |

SNP: Single nucleotide polymorphism; UEP: Unextended mini sequencing primer

Supplementary **Table 2. The information and HWE about the candidate SNPs in *LRRC3B***

| **SNP ID** | **Chr:Position** | **Role** | **Alleles**  **(A/B)** | **MAF** | | ***p*-value for HWE** | **Haploreg** |
| --- | --- | --- | --- | --- | --- | --- | --- |
|  |  |  |  | **Cases** | **Controls** |  |  |
| rs112276562 | 3:26637037 | intronic | C/T | 0.19 | 0.20 | 0.892 | Motifs changed |
| rs6790894 | 3:26640868 | intronic | C/T | 0.18 | 0.17 | 0.652 | Enhancer histone marks, Motifs changed, GRASP QTL hits |
| rs6551121 | 3:26652784 | intronic | A/G | 0.49 | 0.49 | 0.799 | Enhancer histone marks, Motifs changed |
| rs6551122 | 3:26652973 | intronic | A/T | 0.38 | 0.35 | 1.000 | Enhancer histone marks, Motifs changed |
| rs1907168 | 3:26658680 | intronic | A/T | 0.12 | 0.15 | 0.138 | Enhancer histone marks, Motifs changed |
| rs73150416 | 3:26661601 | intronic | C/T | 0.18 | 0.17 | 0.766 | Motifs changed |
| rs12635768 | 3:26664745 | intronic | C/T | 0.24 | 0.25 | 0.111 | Motifs changed |
| rs6551130 | 3:26680648 | intronic | C/T | 0.50 | 0.48 | 0.932 | Enhancer histone marks, Motifs changed, GRASP QTL hits |
| rs78205284 | 3:26692758 | intronic | G/T | 0.20 | 0.18 | 0.660 | Motifs changed |
| rs6788033 | 3:26694810 | intronic | C/T | 0.23 | 0.23 | 0.276 | Motifs changed, GRASP QTL hits |

HWE, Hardy-Weinberg equilibrium; SNP, single nucleotide polymorphism; MAF, minor allele frequency; eQTL, expression quantitative trait loci.

Supplementary Table 3. Relationships between *LRRC3B* polymorphisms and **breast cancer** risk

| SNP ID | Model | Genotype | Case | Control | Adjusted by age and gender | |
| --- | --- | --- | --- | --- | --- | --- |
|  |  |  |  |  | OR (95%CI) | p |
| rs112276562 | Allele | C | 905 | 885 | 1.00 | 0.971 |
|  |  | T | 219 | 215 | 1.00 (0.81-1.23) |  |
|  | Genotype | CC | 366 | 355 | 1.00 |  |
|  |  | CT | 173 | 175 | 0.95 (0.74-1.23) | 0.703 |
|  |  | TT | 23 | 20 | 1.12 (0.60-2.08) | 0.719 |
|  | Dominant | CC | 366 | 355 | 1.00 | 0.801 |
|  |  | CT-TT | 196 | 195 | 0.97 (0.76-1.24) |  |
|  | Recessive | CC-CT | 539 | 530 | 1.00 | 0.679 |
|  |  | TT | 23 | 30 | 1.14 (0.62-2.10) |  |
|  | Log-additive | --- | --- | --- | 0.99 (0.80-1.22) | 0.942 |
| rs6790894 | Allele | T | 918 | 915 | 1.00 | 0.404 |
|  |  | C | 208 | 189 | 1.10 (0.88-1.36) |  |
|  | Genotype | TT | 373 | 377 | 1.00 |  |
|  |  | TC | 172 | 161 | 1.08 (0.84-1.40) | 0.543 |
|  |  | CC | 18 | 14 | 1.31 (0.64-2.68) | 0.455 |
|  | Dominant | TT | 373 | 377 | 1.00 | 0.449 |
|  |  | TC-CC | 190 | 174 | 1.10 (0.86-1.42) |  |
|  | Recessive | TT-TC | 545 | 538 | 1.00 | 0.494 |
|  |  | CC | 18 | 14 | 1.28 (0.63-2.61) |  |
|  | Log-additive | --- | --- | --- | 1.10 (0.89-1.38) | 0.380 |
| rs6551121 | Allele | G | 568 | 557 | 1.00 | 0.996 |
|  |  | A | 556 | 545 | 1.00 (0.85-1.18) |  |
|  | Genotype | GG | 136 | 139 | 1.00 |  |
|  |  | GA | 296 | 279 | 1.09 (0.82-1.46) | 0.553 |
|  |  | AA | 130 | 133 | 1.01 (0.72-1.41) | 0.967 |
|  | Dominant | GG | 136 | 139 | 1.00 | 0.656 |
|  |  | GA-AA | 426 | 412 | 1.06 (0.81-1.40) |  |
|  | Recessive | GG-GA | 432 | 418 | 1.00 | 0.715 |
|  |  | AA | 130 | 133 | 0.95 (0.72-1.25) |  |
|  | Log-additive | --- | --- | --- | 1.01 (0.85-1.19) | 0.958 |
| rs6551122 | Allele | A | 697 | 714 | 1.00 | 0.157 |
|  |  | T | 429 | 388 | 1.13 (0.95-1.35) |  |
|  | Genotype | AA | 210 | 231 | 1.00 |  |
|  |  | AT | 277 | 252 | 1.20 (0.93-1.55) | 0.150 |
|  |  | TT | 76 | 68 | 1.23 (0.84-1.79) | 0.281 |
|  | Dominant | AA | 210 | 231 | 1.00 | 0.121 |
|  |  | AT-TT | 353 | 320 | 1.21 (0.95-1.54) |  |
|  | Recessive | AA-AT | 487 | 483 | 1.00 | 0.553 |
|  |  | TT | 76 | 68 | 1.11 (0.78-1.58) |  |
|  | Log-additive | --- | --- | --- | 1.14 (0.95-1.35) | 0.155 |
| rs73150416 | Allele | T | 923 | 911 | 1.00 | 0.733 |
|  |  | C | 201 | 191 | 1.04 (0.84-1.29) |  |
|  | Genotype | TT | 383 | 375 | 1.00 |  |
|  |  | TC | 157 | 161 | 0.95 (0.73-1.23) | 0.690 |
|  |  | CC | 22 | 15 | 1.44 (0.73-2.81) | 0.292 |
|  | Dominant | TT | 383 | 375 | 1.00 | 0.936 |
|  |  | TC-CC | 179 | 176 | 0.99 (0.77-1.27) |  |
|  | Recessive | TT-TC | 540 | 536 | 1.00 | 0.268 |
|  |  | CC | 22 | 15 | 1.46 (0.75-2.84) |  |
|  | Log-additive | --- | --- | --- | 1.03 (0.83-1.28) | 0.765 |
| rs12635768 | Allele | C | 860 | 828 | 1.00 | 0.449 |
|  |  | T | 266 | 276 | 0.93 (0.76-1.13) |  |
|  | Genotype | CC | 328 | 303 | 1.00 |  |
|  |  | CT | 204 | 222 | 0.85 (0.67-1.09) | 0.199 |
|  |  | TT | 31 | 27 | 1.07 (0.62-1.83) | 0.810 |
|  | Dominant | CC | 328 | 303 | 1.00 | 0.268 |
|  |  | CT-TT | 235 | 249 | 0.87 (0.69-1.11) |  |
|  | Recessive | CC-CT | 532 | 525 | 1.00 | 0.629 |
|  |  | TT | 31 | 27 | 1.14 (0.67-1.94) |  |
|  | Log-additive | --- | --- | --- | 0.93 (0.76-1.13) | 0.458 |
| rs6551130 | Allele | T | 558 | 573 | 1.00 | 0.232 |
|  |  | C | 568 | 527 | 1.11 (0.94-1.31) |  |
|  | Genotype | TT | 139 | 150 | 1.00 |  |
|  |  | TC | 280 | 273 | 1.11 (0.84-1.48) | 0.459 |
|  |  | CC | 144 | 127 | 1.23 (0.88-1.72) | 0.216 |
|  | Dominant | TT | 139 | 150 | 1.00 | 0.303 |
|  |  | TC-CC | 424 | 400 | 1.15 (0.88-1.51) |  |
|  | Recessive | TT-TC | 419 | 423 | 1.00 | 0.321 |
|  |  | CC | 144 | 127 | 1.15 (0.87-1.51) |  |
|  | Log-additive | --- | --- | --- | 1.11 (0.94-1.31) | 0.216 |
| rs78205284 | Allele | G | 901 | 905 | 1.00 | 0.202 |
|  |  | T | 223 | 195 | 1.15 (0.93-1.42) |  |
|  | Genotype | GG | 370 | 374 | 1.00 |  |
|  |  | GT | 161 | 157 | 1.04 (0.80-1.35) | 0.770 |
|  |  | TT | 31 | 19 | 1.66 (0.92-2.99) | 0.093 |
|  | Dominant | GG | 370 | 374 | 1.00 | 0.428 |
|  |  | GT-TT | 192 | 176 | 1.11 (0.86-1.42) |  |
|  | Recessive | GG-GT | 531 | 513 | 1.00 | 0.098 |
|  |  | TT | 31 | 19 | 1.64 (0.91-2.94) |  |
|  | Log-additive | --- | --- | --- | 1.14 (0.93-1.41) | 0.207 |
| rs6788033 | Allele | C | 871 | 853 | 1.00 | 0.960 |
|  |  | T | 255 | 251 | 0.99 (0.82-1.21) |  |
|  | Genotype | CC | 333 | 334 | 1.00 |  |
|  |  | CT | 205 | 185 | 1.12 (0.87-1.43) | 0.389 |
|  |  | TT | 25 | 33 | 0.76 (0.44-1.31) | 0.328 |
|  | Dominant | CC | 333 | 334 | 1.00 | 0.618 |
|  |  | CT-TT | 230 | 218 | 1.06 (0.84-1.35) |  |
|  | Recessive | CC-CT | 538 | 519 | 1.00 | 0.254 |
|  |  | TT | 25 | 33 | 0.73 (0.43-1.25) |  |
|  | Log-additive | --- | --- | --- | 1.00 (0.82-1.22) | 0.986 |

SNP, single nucleotide polymorphism; OR, odds ratio; 95% CI, 95% confidence interval.

*p* values were calculated by logistic regression analysis with adjustments for age and gender.

*p* < 0.05 means the data is statistically significant.

Supplementary **Table 4. Haplotype frequencies of *LRRC3B* gene and their associations with breast cancer risk**

| **Block** | **SNP** | **Haplotype** | **Frequency** | | **Adjusted by age and gender** | |
| --- | --- | --- | --- | --- | --- | --- |
|  |  |  | **Case** | **Control** | **OR (95% CI)** | ***p*** |
| Block 1 | rs112276562\|rs6790894 | CC | 0.81 | 0.83 | 0.90 (0.72-1.12) | 0.358 |
|  | rs112276562\|rs6790894 | TT | 0.81 | 0.80 | 1.01 (0.82-1.24) | 0.942 |
|  | rs112276562\|rs6790894 | CT | 0.62 | 0.63 | 0.94 (0.79-1.12) | 0.502 |
| Block 2 | rs6551121\|rs6551122\|rs1907168\|rs73150416 | GTAC | 0.82 | 0.83 | 0.95 (0.76-1.19) | 0.665 |
|  | rs6551121\|rs6551122\|rs1907168\|rs73150416 | GATT | 0.88 | 0.85 | **1.29 (1.00-1.65)** | **0.048** |
|  | rs6551121\|rs6551122\|rs1907168\|rs73150416 | GTAT | 0.20 | 0.18 | 1.16 (0.93-1.44) | 0.181 |
|  | rs6551121\|rs6551122\|rs1907168\|rs73150416 | AAAT | 0.49 | 0.49 | 1.02 (0.86-1.20) | 0.853 |
| Block 3 | rs12635768\|rs6551130\|rs78205284\|rs6788033 | CTGT | 0.78 | 0.77 | 1.03 (0.84-1.26) | 0.792 |
|  | rs12635768\|rs6551130\|rs78205284\|rs6788033 | CCTC | 0.20 | 0.18 | 1.15 (0.93-1.41) | 0.195 |
|  | rs12635768\|rs6551130\|rs78205284\|rs6788033 | TCGC | 0.23 | 0.25 | 0.93 (0.76-1.13) | 0.461 |
|  | rs12635768\|rs6551130\|rs78205284\|rs6788033 | CCGC | 0.93 | 0.95 | 0.72 (0.50-1.03) | 0.068 |
|  | rs12635768\|rs6551130\|rs78205284\|rs6788033 | CTGC | 0.73 | 0.70 | 1.13 (0.94-1.36) | 0.200 |

OR: odds ratio; 95% CI: 95% confidence interval. *p* values were calculated using logistic regression analysis with adjustment by gender and age; *p* < 0.05 indicates statistical significance.
